# Electric Arc Metallothermic Smelting of FeCr Using FeAlSiCa as a Reductant

**DOI:** 10.3390/ma18184221

**Published:** 2025-09-09

**Authors:** Yerbolat Makhambetov, Zhadiger Sadyk, Armat Zhakan, Azamat Burumbayev, Sultan Kabylkanov, Aibar Myrzagaliyev, Dastan Aubakirov, Natalya Lu, Amankeldy Akhmetov

**Affiliations:** 1 Zh. Abishev Chemical-Metallurgical Institute, Ermekov Street 63, Karaganda 100009, Kazakhstan; makhambetovyerbolat@gmail.com (Y.M.); armat.medetuly@gmail.com (A.Z.); burumbayev.azamat@mail.ru (A.B.); kabyl_96@mail.ru (S.K.);; 2 ERG Research and Engineering Center LLP, Kunayev Street 2, Astana 010000, Kazakhstan; aibar.myrzagaliyev@erg.kz; 3 Department of Nanotechnology and Metallurgy, Abylkas Saginov Karaganda Technical University, Nazarbayev Avenue, No. 56, Karaganda 100027, Kazakhstan; 4 QazMetals Engineering, Mukanov Street 61/2, 198, Karaganda 100024, Kazakhstan

**Keywords:** ferrochrome, chromium, aluminum, silicon, calcium, metallothermy, metallurgy, sustainability

## Abstract

This study investigates the use of the complex reductant FeAlSiCa as an alternative to the conventional FeSiCr in the EAF smelting of FeCr. The smelting process using FeAlSiCa is characterized by a stable furnace operation, active discharge of metal and slag, and effective phase separation. It was found that a 20% excess of FeAlSiCa over the stoichiometric requirement leads to a sharp increase in Si content in the FeCr alloy, with approximately 85% Cr recovery into the metal. A stoichiometric amount of FeAlSiCa results in a metal with 1.5–1.6% Si content and about 80% Cr recovery. A comparable Cr recovery using FeSiCr was achieved only when applying a 20% excess of that reductant. The use of FeAlSiCa also holds promise for technological sustainability due to its low production cost and the utilization of waste materials during its synthesis. The resulting slags are solid and rock-like and show no signs of disintegration after storage for more than 45 days.

## 1. Introduction

Traditionally, FeCr is produced by smelting chromite ore or concentrate in an EAF [1,2,3]. EAF is the dominant unit in industrial FeCr production, where the charge is heated by electric arcs, usually struck between carbon electrodes and the burden. Such furnaces operate at very high temperatures (exceeding 1700–1800 °C), allowing efficient reduction in Cr oxides with carbonaceous reductants (coke, coal, or char) and the formation of a FeCr alloy. EAFs are widely used due to their flexibility in raw material usage, relatively low capital costs compared to blast furnaces, and their ability to process fine ores and concentrates.

Depending on the desired grade of FeCr, different types of reductants are employed. For low-carbon (LC) FeCr, the process generally involves silicothermic reduction, and less frequently, aluminothermic methods are used. This preference is largely due to the lower cost of Si sources and the ease of introducing Si into the melt.

Si is typically added in the form of FeSiCr, while Al is more often introduced as secondary Al scrap. If there is no Al production facility nearby or no easy access to recycled Al, Si becomes the only practical reductant, since it can be locally produced in quantities sufficient to support LC FeCr production [4,5].

Considering efforts to reduce environmental impacts and greenhouse gas emissions associated with LC FeCr production, the search for alternative reductants has become increasingly relevant.

FeSiCr is produced via high-temperature carbothermic reduction in chromite and silica using carbonaceous fuels (such as coke or coal) in electric arc furnaces. This process results in significant CO_2_ emissions. The carbon footprint of producing 1 t of FeSiCr reductant can amount to several t of CO_2_ equivalent, depending on the technology and energy sources used. The main contributors to emissions are the oxidation of carbon (with a mass ratio of 3.67 CO_2_/C) during oxide reduction and the electricity consumption in the furnace, which are widely recognized as the primary sources of greenhouse gas emissions in metallurgical processes. For instance, the production of FeSi—a process similar to the reduction in SiO_2_—under clean energy conditions (e.g., in Australia) results in emissions of about 3.4 t CO_2_ per t of alloy [6]. However, under fossil fuel-based power generation (primarily coal-fired), the carbon footprint is substantially higher. For metallurgical-grade Si (nearly 100% Si), total emissions can reach 10–12 t CO_2_ per t of Si, with more than half resulting from electricity generated by fossil fuel combustion, and the remainder originating from direct CO_2_ emissions during the carbothermic reduction in silica [7]. For high-carbon FeCr, the carbon footprint is estimated in the range of 1.8–5.5 t CO_2_ per t of alloy, again depending on the electricity source [8]. Based on these data, the overall carbon footprint of FeSiCr can be roughly estimated at 8–10 t CO_2_-equivalent per t of reductant when fossil-based electricity is used. Approximately 40% of these emissions result from the carbothermic reduction in Cr_2_O_3_ and SiO_2_ to alloy (with release of CO/CO_2_).

Currently, there are virtually no widely adopted industrial alternatives for LC FeCr smelting. Experimental studies have demonstrated the effectiveness of using Si-rich cutting waste from the solar wafer cutting process [9], highlighting the potential of utilizing various waste streams to lower the production cost and environmental impact of FeCr. However, such waste requires thorough preprocessing to extract material suitable for use as a reductant [9,10,11]. In the case of secondary Al, the main issue is its high oxidation loss during melting, which in industrial settings reaches 72–78% [12,13]. This necessitates the use of Al in the form of ferroalloys, which can improve assimilation into the metal and reduce oxidation losses [14].

Previous studies have demonstrated the feasibility of producing LC FeCr using FeAlSiCa [15,16]—a complex ferroalloy derived from high-ash coal and blast furnace slag, which contributes to its low cost. The average composition of FeAlSiCa is as follows (in wt.%): 17–24 Al, 42–53 Si, 8–15 Ca, and the balance Fe. The main components of this ferroalloy produce an exothermic effect during the reduction of Cr_2_O_3_, which positively affects the energy efficiency of the process [17].

In earlier research [15], it was found that a 10% excess of FeAlSiCa during crucible smelting of Cr concentrate in a chamber furnace led to high Cr recovery into the metal (86%) while avoiding excessive transfer of Si. The objective of the present work is to expand these studies to a larger laboratory scale using an electric arc furnace to smelt FeCr with FeAlSiCa as the reductant.

## 2. Materials and Methods

The source of Cr used in this study was a Cr concentrate (its chemical composition is presented in Table 1). The reductant was FeAlSiCa (composition shown in Table 2), and lime was added with the following specifications: CaO ≥ 92%, SiO_2_ ≤ 1.8%, S ≤ 0.03%, P ≤ 0.02%, and calcination losses ≤ 6%.

FeAlSiCa was introduced in both a 20% excess relative to the stoichiometric requirement and at the exact stoichiometric amount. Lime was added in a calculated ratio of 1.8 parts CaO per 1 part SiO_2_ formed during smelting, with the aim of binding Si, S, and P.

To compare the efficiency of FeAlSiCa as a reductant, a parallel smelting trial was conducted under similar conditions using the conventional FeSiCr alloy, whose composition is provided in Table 3.

The chemical compositions presented in Table 1, Table 2 and Table 3 were determined using standard analytical chemistry methods. Analysis of the Cr concentrate was performed based on GOST 15848.0–90 (ISO 6629–81) [18]. The compositions of FeAlSiCa and FeSiCr alloys were determined based on the procedures specified in GOST 11861–91 (ISO 5449–8) [19], which regulates the analysis of FeSiCr alloys. In the absence of a dedicated standard for FeAlSiCa, the methods established for FeCr and FeSiCr were applied as the closest equivalents.

FeSiCr was similarly introduced with a 20% excess over the stoichiometric amount, and the lime addition was also calculated at a ratio of 1.8 parts CaO per 1 part SiO_2_.

The stoichiometric quantities of reductants were calculated based on the total oxygen content in the oxide components of the charge, primarily contributed by Cr_2_O_3_, Fe_2_O_3_, SiO_2_, Al_2_O_3_, and MgO. The oxygen balance was established using classical thermochemical reduction reactions (Si → SiO_2_, Ca → CaO, Al → Al_2_O_3_). For each melt, the amount of FeAlSiCa reductant was calculated such that the combined molar contribution of Si, Al, and Ca would be sufficient to reduce the available oxygen in the charge to the corresponding oxides. The calculation considered the specific composition of the FeAlSiCa alloy (mass fractions of active elements, Table 2) and their respective oxygen-reducing capacities.

Analogous stoichiometric calculations were performed for the FeSiCr reductant, based primarily on the Si content responsible for oxygen removal. The required amount of FeSiCr was estimated so that the available Si would match the stoichiometric oxygen demand of the charge.

According to these calculations, the stoichiometric amount of FeAlSiCa required to reduce 100 kg of Cr concentrate was 32 kg, increasing to 38 kg when a 20% excess was applied. For FeSiCr, the corresponding values were 46 kg and 55 kg, respectively.

For FeAlSiCa, the expected Cr content in the FeCr alloy was approximately 69% under stoichiometric conditions and decreased to about 65.5% with a 20% excess of the reductant due to dilution by increased Si content. The expected Cr content in FeCr when using FeSiCr as the reductant was approximately 73.2% under stoichiometric conditions and decreased to about 70.2% with a 20% excess, accompanied by a significant increase in Si content.

**Figure 1:**
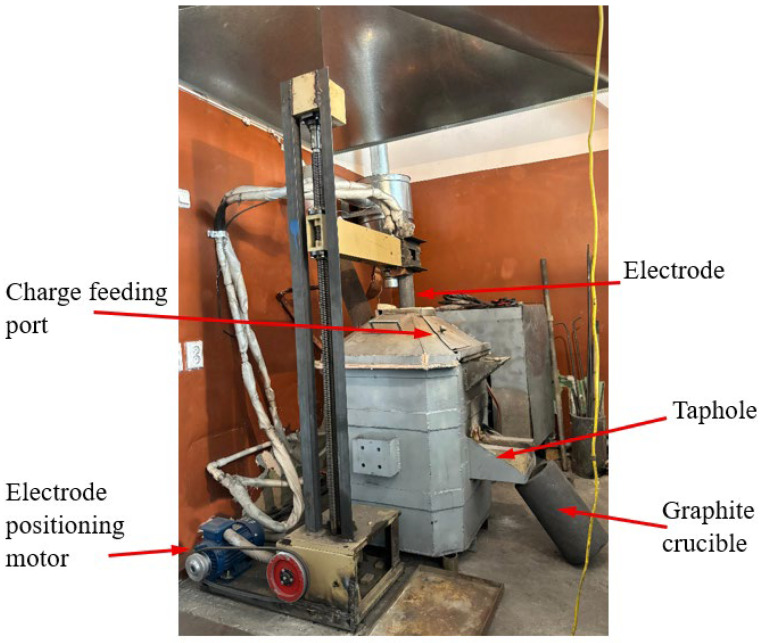
The external view of the furnace.

The primary equipment used for the smelting experiments was a laboratory-scale electric arc furnace (custom-built by the authors, Zh. Abishev Chemical-Metallurgical Institute Karaganda, Kazakhstan) with a transformer power rating of 80 kVA. Figure 1 shows the external view of the furnace.

The electric arc furnace used in this study was equipped with a single graphite electrode with a diameter of 100 mm. To ensure experimental purity and prevent Cr_2_O_3_ contamination from the refractory lining, the furnace was lined with MP-95 grade magnesite refractory bricks, and the joints were filled with magnesite powder. The working volume of the furnace bath was approximately 15 L. All experiments were carried out under ambient air atmosphere without the use of protective gases or vacuum, which corresponds to conventional conditions of ferroalloy smelting in open EAFs.

The transformer operating voltage ranged from 14 V to 24 V. The furnace was preheated for 4 h using a coke bed as a conductive medium. After the preheating period, the bath was thoroughly cleaned of any remaining coke. During the preheating phase, the electrical regime was maintained at 14 V, with an average current load on the electrodes ranging from 70 A to 90 A. After preheating, the arc was subsequently established directly between the graphite electrode and the conductive furnace hearth.

The technological process was carried out as follows. A small amount of Cr concentrate was first charged into the preheated furnace bath to initiate current flow under the electrodes. Once a stable current load was established, the calculated charge of Cr concentrate, FeAlSiCa, and lime was added. The first two melts were carried out as flushing heats to develop a protective slag coating; their results were excluded from the overall analysis.

Smelting was conducted by initially charging a measured portion of FeAlSiCa onto the hearth of the furnace. Immediately after arc ignition, part of the batch consisting of Cr concentrate and lime was introduced. Once a stable electrical load was achieved, the remainder of the batch (Cr concentrate and lime) was added. The total burden for each melt consisted of a single charge of the specified composition. A second portion was gradually introduced after a slag layer had formed in the furnace bath. The electrical parameters during the experimental melting phase were 24 V and 80 A. The duration of smelting–from the initial charge to the discharge of metal and slag–was 1.5 h to 2.0 h. Prior to tapping, the furnace was shut off, and the metal and slag were discharged through the tap hole into a graphite crucible. Samples of metal and slag were then collected for chemical analysis.

Each smelting run was carried out using a charge consisting of 4.2 kg of Cr concentrate and either 1.6 kg (with 20% excess) or 1.3 kg (stoichiometric) of FeAlSiCa, or 2.31 kg of FeSiCr (with 20% excess) and ~4 kg lime. The Cr concentrate used in the charge had a particle size of 2–5 mm, while the reductants (FeAlSiCa and FeSiCr) and lime were sized to 5–10 mm.

After tapping, the current loading was resumed either on bulk FeAlSiCa or on the remaining melt in the furnace bath. When loading on bulk FeAlSiCa, Cr concentrate, and lime were added in the calculated proportions to neutralize the reductant.

FeCr smelting trials using FeSiCr were conducted under nearly identical conditions and operating regimes.

The chemical composition of metal and slag samples was determined using analytical chemistry methods, including acid digestion (with mixtures of HCl, HNO_3_, HF, and others as needed), as well as gravimetric and volumetric titration techniques in accordance with the standards referenced in GOST 4757-91 (ISO 5448-81) Ferrochromium. Specification and conditions of delivery [20].

The resulting metal and slag samples were examined using a Zeptools ZEM20 (ZEPTOOLS, Tongling City, China) scanning electron microscope (SEM) equipped with an Oxford energy-dispersive spectroscopy (Oxford Instruments, Abingdon, UK) detector.

A simplified flow of the experimental procedure can be summarized as follows: charge preparation → furnace preheating → smelting → tapping of metal and slag → sampling and chemical analysis. All experiments were conducted under atmospheric pressure in an air atmosphere, with smelting temperatures exceeding 1650–1700 °C.

## 3. Results

### 3.1. Smelting Process

During the smelting runs using FeAlSiCa, metal was discharged actively and vigorously with characteristic sparking typical for Cr-containing ferroalloys, and the slag exhibited good fluidity. Overall, the FeCr smelting process with the new reductant was marked by stable electrode positioning. The use of FeAlSiCa led to process intensification inside the furnace, and under such intensified conditions, the electrical regime remained stable with a consistent current load. Fluctuations in current were observed approximately 1–1.5 h after tapping, as metal accumulated in the furnace. After tapping, cleaning of the burden residue, and charging of a new batch, the current load stabilized again. The charged materials were uniformly heated by the outgoing reaction gases, creating favorable conditions for reduction reactions. No significant deviations from normal operating conditions were observed. The reaction zone reached white heat, and molten metal flowed actively through the tapping channel. When necessary, stirring was performed manually using steel rods.

The smelting process using FeSiCr was carried out following an intermediate smelt, the results of which were not included in this study. The main smelting trials were then conducted under nearly identical conditions. This process also demonstrated expected stability, with active metal discharge; however, the slag was noticeably more viscous under the same electrical load. In several runs, the slag appeared thick and sticky, requiring manual removal. This is due to the fact that in the case of FeSiCr, Si is essentially the sole reductant. As a result, its higher content in the charge led to the formation of a more silica-rich slag with a high SiO_2_ content. Such slag is acidic and, without sufficient dilution by basic oxides, has high viscosity and relatively low electrical conductivity [21]. An excess of SiO_2_ leads to slag polymerization, which further reduces its fluidity and electrical conductivity [22].

In contrast, when using FeAlSiCa, the slag composition was initially more basic due to higher levels of Ca and Al. The oxides of Ca and Al significantly improve the electrical conductivity of the melt and reduce its specific electrical resistance. This contributes to more stable arc operation (the arc burns more steadily and is less sensitive to compositional fluctuations). Moreover, the expected more energetically intense exothermic reactions locally maintain higher temperatures. As a result, the electric arc process using FeAlSiCa exhibits enhanced stability: the arc remains steady, less dependent on external adjustments, as the slag conducts current better and in situ heat generation mitigates power fluctuations. In contrast, maintaining arc stability in FeSiCr-based smelting requires stricter control of the SiO_2_/CaO ratio in the slag and continuous arc current regulation.

Aluminothermic reactions are known for their ability to heat the charge to extremely high temperatures (>2000 °C), enabling the melting of ore and slag-forming components with minimal support from the arc. Consequently, the process proceeds steadily with a reduced risk of freeze-up of the molten bath.

At approximately 1600 °C, the reduction reactions of Cr_2_O_3_ by the main active elements in FeAlSiCa proceed as follows [23]:

Cr_2_O_3_ (s) + 2Al (l) → 2Cr (l) + Al_2_O_3_ (s) (ΔG°_1873K_ ≈ −496 kJ/mol) (1)

Cr_2_O_3_ (s) + 3Si (l) → 2Cr (l) + 3SiO_2_ (l) (ΔG°_1873K_ ≈ −191 kJ/mol) (2)

Cr_2_O_3_ (s) + 3Ca (l) → 2Cr (l) + 3CaO (s) (ΔG°_1873K_ ≈ −620 kJ/mol) (3)

As these thermodynamic data indicate, Al and Ca reduce Cr_2_O_3_ in significantly more exothermic reactions compared to Si, which is essentially the only active reductant in traditional FeSiCr. Therefore, when FeSiCr is used alone, the reaction temperature primarily depends on the electric arc power. The furnace must compensate for the lack of thermal energy by increasing power input. In practice, this challenge is sometimes addressed by feeding molten FeSiCr (at ~1550 °C) into the reaction zone [22] or by applying prolonged heating of the solid charge to ensure complete melting. Thus, in FeSiCr-based processes, the temperature profile is largely governed by the furnace operation, whereas with FeAlSiCa, a significant portion of the heat is generated in situ, stabilizing the thermal regime.

### 3.2. Investigation of Smelting Products

**Figure 2:**
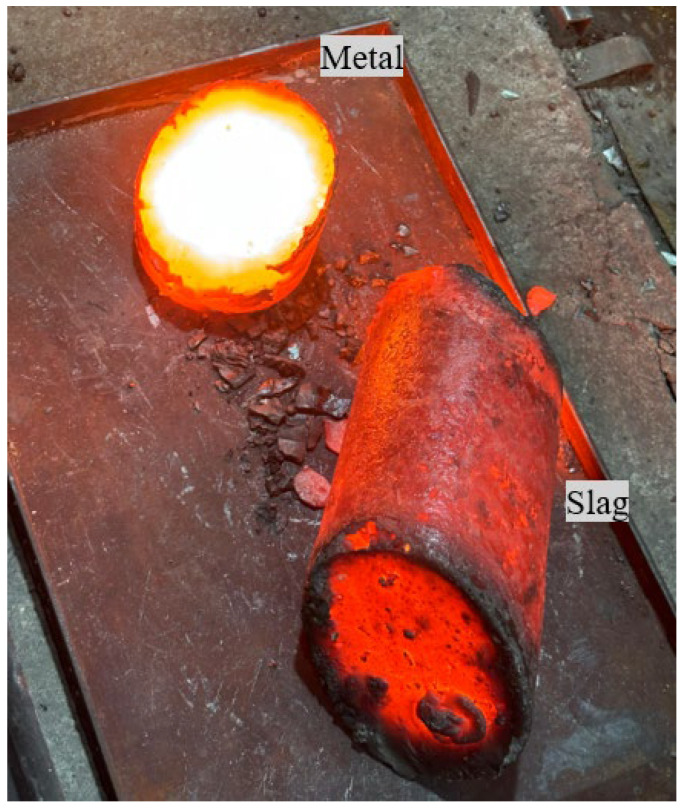
Typical appearance of FeCr smelting products.

The products of the smelting process consisted of metal and slag samples. Figure 2 shows the typical appearance of the resulting materials.

As seen in Figure 2, the tapping of the furnace yielded sizable masses of both metal and slag. To determine the chemical composition, samples were collected by crushing and grinding metal fragments taken from different sections of the solidified metal mass. The summarized average results of the analysis are presented in Table 4.

As shown in Table 4, a high Si content (15–18%) was observed when FeAlSiCa was introduced with a 20% excess (Runs 1, 2, and 3). The excess FeAlSiCa was added to compensate for potential oxidation losses when the reductant was exposed to air. When FeAlSiCa was added at the stoichiometric level (Runs 4 and 5), the Si content in the resulting alloy dropped significantly. At the same time, the Cr content in the metal reached 70–74%. All samples exhibited elevated carbon content, averaging 3–4%, which is attributed to the carbonaceous hearth of the furnace. Slightly elevated phosphorus levels were also observed, while sulfur content remained within acceptable limits.

In the trials using FeSiCr with a 20% excess, the resulting metal contained a moderate amount of Si, generally within standard specifications. However, the carbon content was significantly higher, due to additional carbon originating from FeSiCr itself, which contains approximately 2.00% C. The phosphorus content was slightly lower, while the sulfur content remained comparable.

To fully assess the effectiveness of FeAlSiCa, a detailed analysis of slag samples—collected in a manner analogous to that used for the metal samples—was also conducted. The results are shown in Table 5.

As shown in Table 5, the residual Cr_2_O_3_ content in slags produced using FeAlSiCa did not exceed 4% when the reductant was used in 20% excess and remained below 6% when used at stoichiometric levels. In contrast, slags from the FeSiCr trials (20% excess) exhibited an average residual Cr_2_O_3_ content of about 8%. The 16% value observed in Run 6 is attributed to the furnace being newly started during this trial, with the reaction zone not yet fully heated. Similarly, Run 9 also showed an elevated Cr_2_O_3_ content (14.87%), which deviates from the general trend and is therefore not considered representative of steady-state operation. Therefore, Runs 7 and 8 are more representative of steady-state conditions.

In the reduction in chromite with FeSiCr, the primary slag component is silica (SiO_2_). The slag also contains oxides originally present in the ore (e.g., MgO, Al_2_O_3_) and flux-derived CaO, along with residual Cr_2_O_3_ from incomplete reduction. This type of slag is acidic (rich in SiO_2_). In contrast, the use of FeAlSiCa results in a greater proportion of Cr being reduced by Al and Ca, leading to higher contents of Al_2_O_3_ and CaO in the slag. As a result, the slag tends to be more basic: in Runs 1, 2, 3, and 4, the basicity index was 1.0, 1.0, 0.8, 0.8, and 0.9, respectively. For Runs 6, 7, 8, and 9, the basicity index was 0.7, 0.9, 0.9, and 1.1, respectively—indicating a relatively small difference. This is due to the introduction of sufficient flux, which allowed for optimal basicity levels typical for low-carbon FeCr production (0.8–1.4), and significantly higher than those observed in high-carbon FeCr slags (~0.5) [24,25,26].

Although increasing basicity through lime addition may enhance Cr recovery, it also leads to higher slag volumes. This can result in greater immersion of the electrode into the molten slag, causing increased electrode wear and arc instability. While higher slag conductivity can be beneficial for energy transfer, the associated increase in slag volume may negatively affect furnace performance [27,28].

Thus, the slags formed with FeAlSiCa were only slightly more basic compared to those obtained with FeSiCr, with basicity indices remaining within the range typical for LC FeCr production. This minor shift toward higher basicity was sufficient to facilitate somewhat better Cr recovery without fundamentally changing the overall slag character.

Slags produced using both types of reductants did not undergo disintegration or self-pulverization after more than 45 days of storage. The slag-to-metal mass ratio averaged 2.5–3.

**Figure 3:**
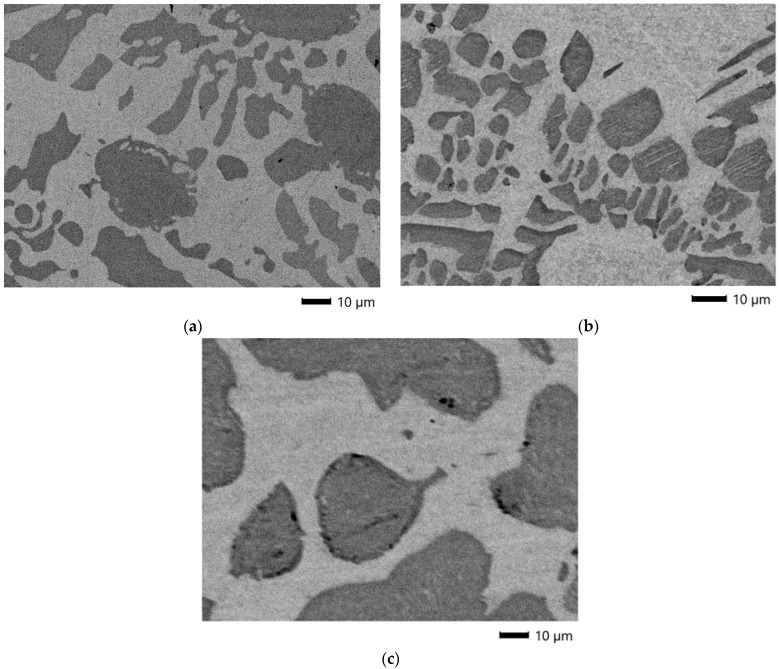
SEM micrographs of FeCr samples produced using: (**a**) 20% excess FeAlSiCa; (**b**) stoichiometric amount of FeAlSiCa; (**c**) 20% excess FeSiCr.

**Figure 4:**
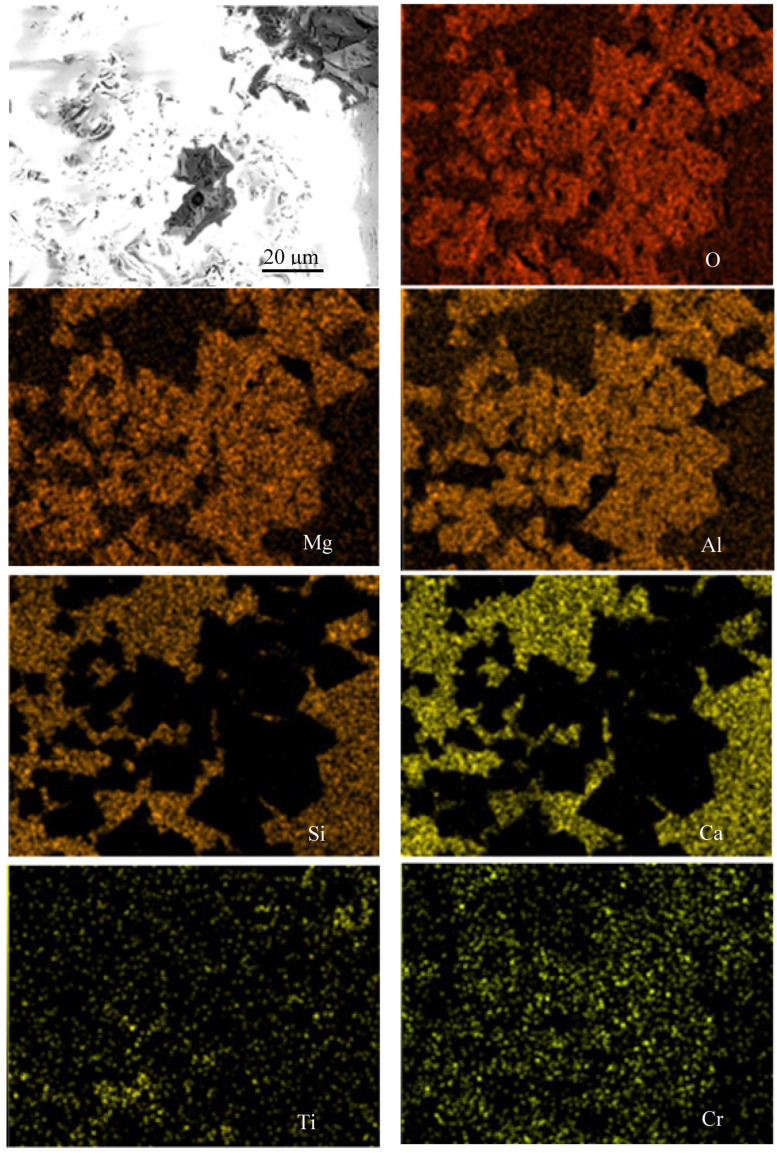
SEM image and elemental distribution map of the slag sample produced using 20% excess FeAlSiCa.

**Figure 5:**
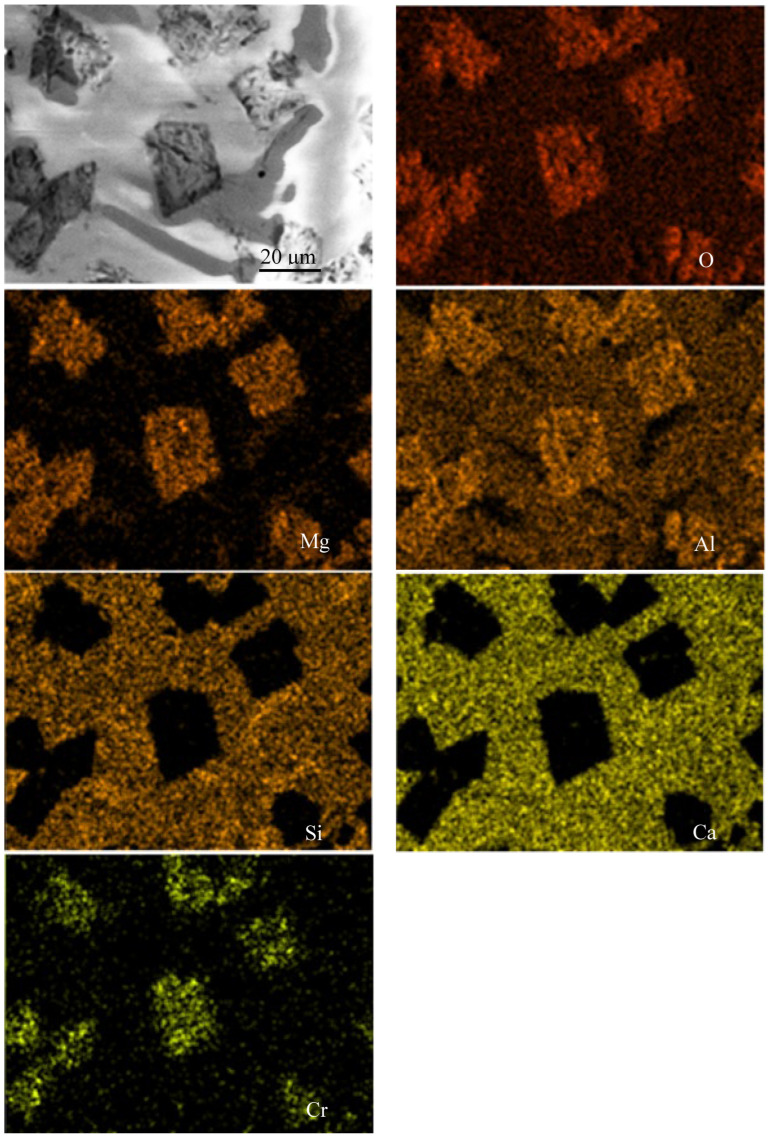
SEM image and elemental distribution map of the slag sample produced using a stoichiometric amount of FeAlSiCa.

**Figure 6:**
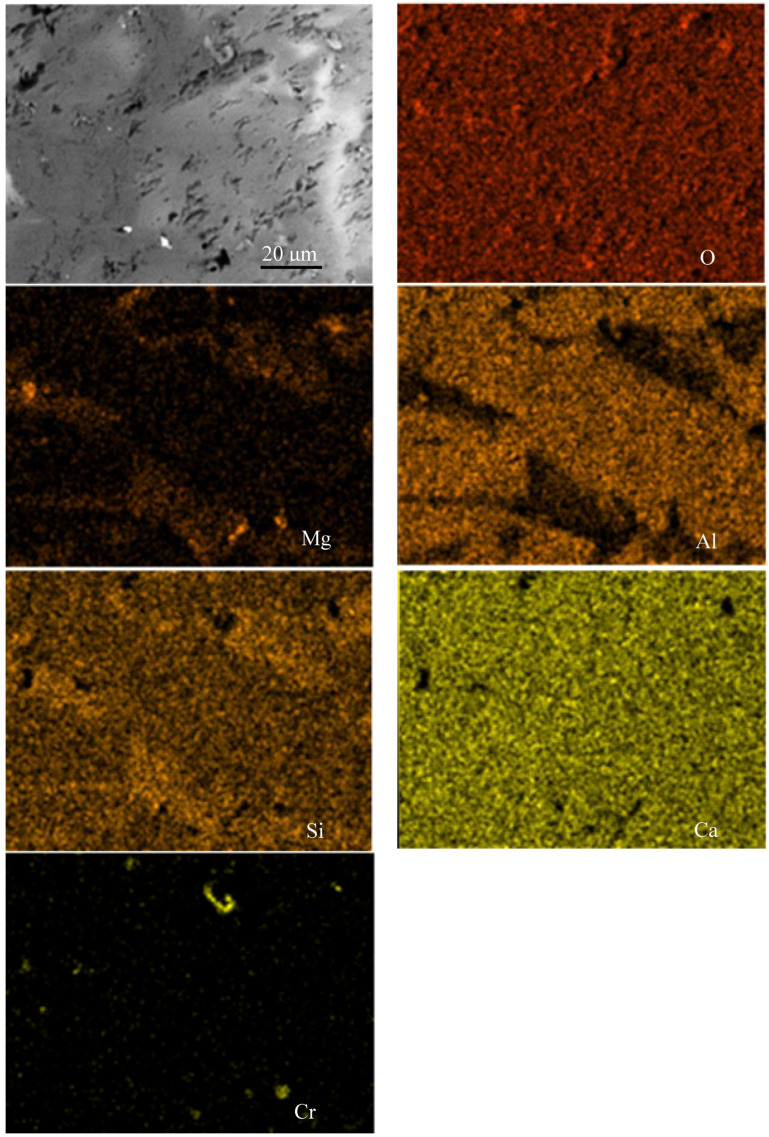
SEM image and elemental distribution map of the slag sample produced using 20% excess FeSiCr.

A study was conducted on representative samples of metal (Figure 3) and slag (Figure 4, Figure 5 and Figure 6) using SEM.

As shown in Figure 3, when the Si content in the alloy is high, the microstructure primarily consists of Cr silicides and a Fe-Cr solid solution. As the Si concentration decreases, carbon, previously mostly dissolved in the Fe-Cr solid solution, forms Cr carbides.

As shown in Figure 4, Figure 5 and Figure 6, when FeAlSiCa is used, the slag contains phases in the Mg–Al–O and Ca–Si–O systems. Based on previous experiments, it can be inferred that arc melting results in the formation of phases like those observed during crucible melts in a chamber furnace [15], such as MgO·Al_2_O_3_ and CaO·SiO_2_. In contrast, slags produced with FeSiCr contain somewhat different phases, primarily within the Mg–Al–Ca–O and Si–Ca–O systems. A notable feature is the more uniform distribution of Ca and its presence in both types of slag phases. Additionally, elemental mapping confirmed the presence of Ti in the slag, indicating that Ti introduced with the FeAlSiCa reductant is oxidized and transitions into the slag phase during smelting.

**Figure 7:**
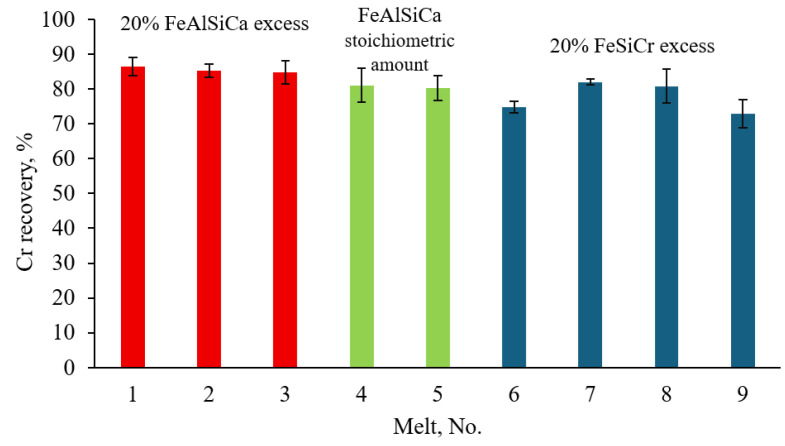
Cr recovery under different smelting conditions.

Based on the chemical composition and mass of the metal samples, Cr recovery rates were calculated. The results are presented in Figure 7.

As shown in Figure 7, the use of FeAlSiCa with a 20% excess enabled average Cr recovery of approximately 85%. When used in stoichiometric amounts, FeAlSiCa resulted in about 80% Cr recovery. The somewhat uneven Cr recovery observed in the FeSiCr trials may be attributed to less stable furnace operation and higher slag viscosity, which can hinder metal-slag separation. On average, Cr recovery using FeSiCr with a 20% excess was approximately 80–82%.

The comparison between the conventional FeSiCr and the complex FeAlSiCa as reductants in FeCr production reveals several promising advantages in favor of the latter. Due to the exothermic nature of aluminothermic and calciothermic reactions, the energy efficiency of the smelting process is improved, and temperature control during melting is facilitated. The higher content of Al and Ca in FeAlSiCa results in greater heat release during reduction compared to FeSiCr, as Si generates less thermal energy in its reduction reactions. In terms of increasing exothermicity of Cr_2_O_3_ reduction reactions, the elements rank as follows: Si < Al < Ca. Practically, this means that the inclusion of Al and especially Ca in the reductant (as in the FeAlSiCa alloy) enhances the contribution of chemical energy to the smelting process compared to the use of a purely Si-based reductant.

The composition of FeAlSiCa promotes the formation of a more fluid and stable slag, which separates efficiently and retains minimal Cr. As a result, the Cr recovery into the metal increases, reducing losses of this valuable element. Economically, this technology is attractive due to its potential for utilizing low-cost secondary raw materials, thereby reducing production costs. From an environmental standpoint, the absence of carbonaceous reductants significantly reduces greenhouse gas emissions and enables the utilization of industrial waste within the complex alloy system.

The metal samples obtained using stoichiometric amounts of FeAlSiCa correspond to the FeCr70C40 grade, with the exception of slightly elevated phosphorus content exceeding the specification of <0.05%. When FeAlSiCa is used in 20% excess, the resulting metal exhibits a significantly higher Si content. For high-carbon FeCr (HC FeCr), some grades require Si <10.0%, while in this study, Si levels reached 15–21%. A similar 20% excess of FeSiCr resulted in FeCr with much higher carbon content (up to 7–10%), but with low Si levels that meet HC FeCr standards. However, these samples also showed elevated phosphorus, which can be attributed to an insufficient amount of flux.

Another important feature of using FeAlSiCa is its influence on slag composition. As previously noted, slag composition and volume play a critical role in determining smelting behavior—affecting temperature profiles, metal-slag separation, and arc stability. In the current process, the presence of Al and Ca in the reductant generates a more basic slag enriched in aluminates and calcium-bearing phases (such as Al_2_O_3_ and CaO·Al_2_O_3_). In contrast, smelting with FeSiCr results in a more acidic slag rich in SiO_2_ and calcium silicates. Slags produced by the latter typically contain dicalcium silicate, which transforms into the γ-form upon cooling, increasing in volume by 10–12% and leading to disintegration into powder [12].

Slag with high SiO_2_ and 2CaO·SiO_2_ content generally has a relatively low liquidus temperature (~1600 °C), which is favorable for FeCr smelting [29]. However, this condition can lead to early-stage “cold furnace operation”, wherein slag melts rapidly at low temperatures, reducing the completeness of Cr reduction [12]. One approach to address this issue is the introduction of boron, which lowers the temperature further, although this risks excessive slag melting. To counteract “cold operation”, lime additions are increased in the charge, which improves Cr recovery but also increases slag volume, electrical conductivity, and ultimately electricity consumption and other techno-economic drawbacks [12,30].

The issue of slag disintegration can be mitigated by using Al as the reductant. However, in addition to cost considerations, slag with high Al_2_O_3_ content (a byproduct of aluminothermy) has a very high melting point and viscosity.

Therefore, the slag phase composition must be carefully tailored to achieve sufficient fluidity at the lowest feasible temperature, without premature melting and slag breakdown. Achieving a basicity ratio of 0.8–1.0 in all representative experiments with both FeAlSiCa and FeSiCr demonstrates the efficiency of these reductants with minimal influence from slag basicity variations.

Another key aspect is the evaluation of greenhouse gas emissions. The metallothermic reductant FeAlSiCa allows for the complete avoidance of CO/CO_2_ emissions during the smelting stage, as most emissions are associated with the production of the reductant itself. As discussed in the Introduction, the use of FeSiCr entails a considerable carbon footprint due to the carbothermic reduction of Si, its main active component. FeAlSiCa carries a similar inherent carbon burden.

In conventional practice, producing a mechanical blend equivalent to FeAlSiCa requires separate stages of Al electrolysis, Si, and CaSi smelting. The key difference is that FeAlSiCa can be synthesized from secondary and low-grade raw materials – blast furnace slag (a primary source of Ca, Si, and Al) and high-ash coal (whose ash provides Si and Al, while its carbon acts as the reductant). This approach helps avoid the additional carbon footprint associated with the preparation of high-grade commercial raw materials and metallurgical coke. Moreover, utilizing these waste materials addresses the environmental challenge of their accumulation and long-term storage.

According to published mass and energy balances [4], conventional FeSiCr-based LC FeCr production processes such as Perrin and Duplex consume ~42 kg of FeSiCr per 100 kg of chromite (~ 49% Cr_2_O_3_) and require 2250–2300 kWh/t LC FeCr. In contrast, proposed aluminothermic and calciothermic technologies demonstrate the potential to significantly reduce both reductant consumption (32 kg per 100 kg of Cr concentrate) and specific energy input.

In addition to thermodynamic and process advantages, FeAlSiCa is significantly more cost-effective than conventional FeSiCr. The FeAlSiCa alloy can be synthesized from industrial waste and secondary raw materials. In contrast, the production of FeSiCr is technologically complex and resource-intensive. It involves either a one-step smelting process—using Cr ore, silica, and coke—or a more elaborate two-step method, which first produces HC FeCr followed by a second reduction stage with silica and coke to form the FeSiCr alloy. Both approaches require high-temperature submerged arc furnaces, large amounts of electrical energy, and tight slag chemistry control to minimize Cr losses and control the final carbon content. The two-step method, in particular, adds operational complexity and additional decarburization steps. These factors result in higher production costs for FeSiCr compared to FeAlSiCa.

## 4. Conclusions

Unlike the previous study [15], which investigated FeCr production using FeAlSiCa in a small-scale crucible furnace, the present work explores arc furnace smelting under conditions closer to industrial practice. In addition, it includes a direct comparison with FeSiCr, phase characterization of slags, and an evaluation of energy and material efficiency.

Electric arc smelting of FeCr using FeAlSiCa as an alternative reductant demonstrated its effectiveness, with high Cr recovery rates of 80–85% into the metal. A 20% excess of the reductant led to a significant increase in Si content in the final alloy, reaching 15–21%, whereas stoichiometric addition resulted in lower Si levels of 1.5–1.6%. Comparative tests using a 20% excess of FeSiCr showed a similar Cr recovery rate of 80–82%.

The lower consumption of FeAlSiCa and its low cost highlight its high potential for application in FeCr production. Stable furnace operation, formation of fluid slag, and easy separation of metal and slag further underscore the process feasibility and technological advantages of this reductant. It is also noteworthy that the resulting slags did not undergo disintegration after more than 45 days of storage.

The presence of elements in FeAlSiCa that reduce Cr_2_O_3_ via more exothermic reactions enhances the energy efficiency of the FeCr production process. Furthermore, the production of FeAlSiCa from blast furnace slag and high-ash coal contributes to reduced reductant costs (and ultimately the cost of FeCr itself) while simultaneously addressing the issue of waste material utilization.

Overall, the use of FeAlSiCa not only resulted in slightly higher and more consistent Cr recovery compared to FeSiCr, but also demonstrated superior energy efficiency due to the strongly exothermic nature of Al- and Ca-based reduction reactions. This confirms that FeAlSiCa can serve as a viable and more sustainable alternative to conventional reductants.

## Figures and Tables

**Table 1:** Chemical composition of Cr concentrate.

Cr_2_O_3_	Al_2_O_3_	SiO_2_	MgO	CaO	Fe_2_O_3_	S	P	CL ^1^
52.91	7.80	4.66	17.5	0.59	12.90	0.023	0.004	3.61

^1^ Calcination loses.

**Table 2:** Chemical composition of FeAlSiCa [15].

Fe	Al	Si	Ca	C	S	P	Ti	V
8.12	13.44	66.15	10.04	0.69	0.018	0.040	1.09	0.41

**Table 3:** Chemical composition of FeSiCr.

Fe	Si	Cr	Al	Ca	C	S	P
9.10	48.07	38.74	4.04	0.018	2.00	0.057	0.024

**Table 4:** Average chemical composition of metal samples.

Reductant	Smelting Run, No.	Fe	Cr	Si	C	P	S
20% excess FeAlSiCa	1	22.0	55.1	18.6	4.25	0.104	0.014
	2	19.8	60.5	15.2	4.44	0.107	0.015
	3	22.4	53.8	21.5	2.14	0.110	0.013
stoichiometric amount of FeAlSiCa	4	20.5	74.3	1.6	3.07	0.064	0.018
	5	23.9	70.7	1.5	3.80	0.068	0.018
20% excess FeSiCr	6	17.4	72.0	2.0	8.61	0.076	0.020
	7	23.6	67.2	1.7	7.38	0.087	0.014
	8	21.1	68.6	0.3	9.93	0.085	0.014
	9	17.5	70.7	0.6	10.98	0.101	0.017

**Table 5:** Chemical composition of slag samples.

Reductant	Smelting Run, No.	Cr_2_O_3_	Fe_2_O_3_	CaO	SiO_2_	Al_2_O_3_	MgO	P	Basicity
FeAlSiCa	1	3.50	0.77	33.44	31.15	16.50	14.64	-	1.0
	2	3.80	1.02	32.84	28.26	17.65	16.42	0.008	1.0
	3	3.24	0.76	29.08	31.89	20.54	14.49	-	0.8
	4	6.03	1.08	26.39	33.18	18.31	14.97	0.043	0.8
	5	5.29	2.27	27.94	32.25	16.18	16.07	-	0.9
FeSiCr	6	16.58	5.15	11.95	27.80	16.48	22.04	0.007	0.7
	7	8.59	2.60	28.36	31.30	14.69	14.46	0.002	0.9
	8	8.09	2.62	29.95	30.30	15.15	13.89	0.003	0.9
	9	14.87	5.29	23.46	22.71	14.03	19.64	0.003	1.1

## Data Availability

The original contributions presented in this study are included in the article. Further inquiries can be directed to the corresponding authors.

## Abbreviations

The following abbreviations are used in this manuscript:

FeCr	Ferrochrome
LC	Low carbon
EAF	Electric arc furnace
SEM	Scanning electron microscope

## References

[1] Shabanov E.Z., Saulebek Z.K., Akhmetov A.S., Mukhtarkhanova G.K. (2024). Smelting of High-Carbon Ferrochromium from Pre-Reduced Chromite Raw Materials. CIS Iron Steel Rev..

[2] Baisanov A., Vorobkalo N., Shabanov Y., Zobnin N., Baisanova A., Sharieva S., Akuov A., Samuratov Y., Ibrakhimova Z., Zhumagaliev T. (2024). Optimization of the Properties of Microsilica-Based Composite Briquettes Depending on Their Granulometry. J. Compos. Sci..

[3] Agarwal S., Pal J., Ghosh D. (2014). Development of Chromite Sinter from Ultra-Fine Chromite Ore by Direct Sintering. ISIJ Int..

[4] Weitz H., Garbers-Craig A.M. (2016). Evaluation of the Furnace Method for the Production of Low Carbon Ferrochrome. Miner. Process. Extr. Metall. Rev..

[5] Lian X., Gao H., Shen L., Yu Y., Wang Y., Peng Z. (2025). Life Cycle Assessment of Primary Aluminum Production. Processes.

[6] Haque N., Norgate T. (2013). Estimation of Greenhouse Gas Emissions from Ferroalloy Production Using Life Cycle Assessment with Particular Reference to Australia. J. Clean. Prod..

[7] Sævarsdottir G., Kvande H., Magnusson T. Greenhouse Gas Emissions from Silicon Production -Development of Carbon Footprint with Changing Energy Systems. Proceedings of the INFACON XVI.

[8] Wei W., Samuelsson P.B., Jönsson P.G., Gyllenram R., Glaser B. (2023). Energy Consumption and Greenhouse Gas Emissions of High-Carbon Ferrochrome Production. JOM.

[9] Sommerfeld M., Weiss J., Friedrich B. (2023). CO_2_-Minimized Ferrochrome Production Utilizing Silicon Wafer Cutting Slurry as an Alternative Reductant. J. Sustain. Metall..

[10] Jung W.-G., Back G.-S., Johra F.T., Kim J.-H., Chang Y.-C., Yoo S.-J. (2018). Preliminary Reduction of Chromium Ore Using Si Sludge Generated in Silicon Wafer Manufacturing Process. J. Min. Metall. B Metall..

[11] Jung W., Hossain S.T., Johra F.T., Kim J., Chang Y. (2019). Reduction of Chromium Ore by Recycled Silicon Cutting Sludge Waste with Carbon Addition. J. Iron Steel Res. Int..

[12] Gilvarg S.I., Odinokov S.F., Bannykh A.G., Kiselev V.M. (2007). Method for Aluminothermic Production of Low-Carbon Ferrochrome.

[13] Zhautykov F.B. (2021). Research and Development of a Two-Stage Steelmaking Process for Smelting, Tapping, and Secondary Metallurgy in a Ladle Furnace Unit for Producing Carbonaceous Semi-Finished Steel. Ph.D. Dissertation.

[14] Akuov A., Samuratov Y., Kelamanov B., Zhumagaliyev Y., Taizhigitova M. (2020). Development of an Alternative Technology for the Production of Refined Ferrochrome. Metalurgija.

[15] Akhmetov A., Zulhan Z., Sadyk Z., Burumbayev A., Zhakan A., Kabylkanov S., Toleukadyr R., Saulebek Z., Ayaganova Z., Makhambetov Y. (2025). Carbon-Free Smelting of Ferrochrome Using FeAlSiCa Alloy. Processes.

[16] Makhambetov Y., Kutzhanov M., Toleukadyr R., Myrzagaliyev A., Sadyk Z., Saulebek Z., Akhmetov A. (2025). Utilization of Chromite Spinel Powder in the Metallothermic Smelting of Low-Carbon Ferrochrome. Processes.

[17] Kirschen M., Badr K., Pfeifer H. (2011). Influence of Direct Reduced Iron on the Energy Balance of the Electric Arc Furnace in Steel Industry. Energy.

[18] (1991). Chromium Ores and Concentrates. General Requirements for Methods of Chemical Analysis.

[19] (2003). Ferrosilicochromium. Specification and Conditions of Delivery.

[20] (2003). Ferrochromium. Specification and Conditions of Delivery.

[21] FSharma L., Chhibber R. (2020). Design & Development of SAW Fluxes Using CaO–SiO_2_–CaF_2_ and CaO–SiO_2_–Al_2_O_3_ Flux Systems. Ceram. Int..

[22] Shoko N.R., Chirasha J. Technological Change Yields Beneficial Process Improvement for Low Carbon Ferrochrome at Zimbabwe Alloys. Proceedings of the Tenth International Ferroalloys Congress (INFACON X).

[23] Akuov A., Tolymbekov M., Kasenov B., Yesenzhulov A. Thermodynamic Analysis of Chrome Reduction with Aluminum and Silicon. Proceedings of the Twelfth International Ferroalloys Congress (INFACON XII).

[24] Horckmans L., Möckel R., Nielsen P., Kukurugya F., Vanhoof C., Morillon A., Algermissen D. (2019). Multi-Analytical Characterization of Slags to Determine the Chromium Concentration for a Possible Re-Extraction. Minerals.

[25] Holappa L., Xiao Y. (2004). Slags in ferroalloys production—Review of present knowledge. J. South. Afr. Inst. Min. Metall..

[26] Harman C.N. A process for the recovery of chromium and iron oxide in high carbon ferro chrome slag to obtain chromium and iron in the form of saleable metal. Proceedings of the INFACON XIII.

[27] Xiao Y., Wei K., Wang L., Liu S., He X., Chou K. (2023). Influence of Slag Chemistry on the Dissolution of FeCr_2_O_4_ in CaO–SiO_2_–Al_2_O_3_–MgO Slag with Graphite Crucible. ISIJ Int..

[28] Jalkanen H., Gasik M. (2013). Theory of Ferroalloys Processing. Handbook of Ferroalloys.

[29] Nakamoto M., Forsbacka L., Holappa L. Assessment of Viscosity of Slags in Ferrochromium Process. Proceedings of the Eleventh International Ferroalloys Congress (INFACON XI).

[30] Salina V.A., Zhuchkov V.I., Zayakin O.V. (2020). Thermodynamic Simulation of Silicothermic Reduction of Chromium. Steel Transl..

